# Intraperitoneal Administration of Etizolam Improves Locomotor Function in Mice After Spinal Cord Injury

**DOI:** 10.1089/neur.2022.0071

**Published:** 2023-02-22

**Authors:** Kenya Saruta, Tatsuhiro Fukutoku, Gentaro Kumagai, Toshihide Nagaoki, Manami Tsukuda, Yohshiro Nitobe, Kanichiro Wada, Toru Asari, Taku Fujita, Isamu Sasaki, Yoshikazu Nikaido, Shuji Shimoyama, Shinya Ueno, Yasuyuki Ishibashi

**Affiliations:** ^1^ Department of Orthopedic Surgery, Hirosaki University Graduate School of Medicine, Hirosaki, Japan.; ^2^ Department of Neurophysiology, Hirosaki University Graduate School of Medicine, Hirosaki, Japan.; ^3^ Department of Anesthesiology, Hirosaki University Graduate School of Medicine, Hirosaki, Japan.

**Keywords:** anxiety, etizolam, functional recovery, GABA_A_ receptor, spinal cord injury (SCI)

## Abstract

Neuroinflammation occurs in the acute phase of spinal cord injury (SCI) and inhibits neural regeneration. In mouse models, etizolam (ETZ) is a strong anxiolytic with unclear effects on SCI. This study investigated the effects of short-term administration of ETZ on neuroinflammation and behavior in mice after SCI. We administrated an ETZ (0.5 mg/kg) daily intraperitoneal injection from the day after SCI for 7 days. Mice were randomly divided into three groups (sham group: only laminectomy, saline group, and ETZ group). Inflammatory cytokine concentrations in the injured spinal cord epicenter were measured using an enzyme-linked immunosorbent assay on day 7 after SCI to evaluate spinal cord inflammation in the acute phase. Behavior analysis was performed the day before surgery and on days 7, 14, 28, and 42 after surgery. The behavioral analysis included anxiety-like behavior using the open field test, locomotor function using the Basso Mouse Scale, and sensory function using the mechanical and heat test. Inflammatory cytokine concentrations were significantly lower in the ETZ group than in the saline group in the acute phase after spinal surgery. After SCI, anxiety-like behaviors and sensory functions were comparable between the ETZ and saline groups. ETZ administration reduced neuroinflammation in the spinal cord and improved locomotor function. Gamma-amino butyric acid type A receptor stimulants may be effective therapeutic agents for patients with SCI.

## Introduction

Spinal cord injury (SCI) causes pain, disability, neuropathy, and affective disorders, such as anxiety and depression. Accumulating studies indicate that the prevalence of anxiety in self-reported measure estimates varies from 15% to 32% and that of depression ranges from 19% to 26%.^1,2^ Affective disorders after SCI inhibit rehabilitation and worsen various physical health problems, such as pneumonia and osteoporosis.^3,4^ They are also associated with adverse outcomes, such as increased incidence of secondary complications,^5^ and the association between anxiety and neuroinflammation in rodent models after SCI has been reported on.^6^ Therefore, treating anxiety disorders plays an essential role in treatment after SCI. However, the effect of anxiolytic drugs on neuroinflammation after SCI remains unclear.

Neuroinflammation, which occurs during the acute and subacute phases of SCI, contributes to tissue damage. Cavities form at the site of tissue injury, and scarring occurs around the cavities, causing permanent motor and sensory dysfunction.^7^ It has been reported that concentrations of inflammatory cytokines were reportedly elevated in the central nervous system (CNS) in a rat SCI model with affective disorders.^6^ In addition, γ-aminobutyric acid (GABA) is a major inhibitory neurotransmitter that regulates the excitability of neurons in the CNS,^8^ and etizolam (ETZ) is a thienodiazepine derivative and a benzodiazepine analog, and it enhances GABAergic activity in neurons through γ-aminobutyric acid type A (GABA_A_) receptors.

Notably, Fujita and colleagues showed that female mice with abnormal GABA_A_ receptor function had increased inflammation after SCI, resulting in anxiety and motor functional disability.^9^ Tian and colleagues reported the anti-inflammatory effects of GABA_A_ receptor agonists in a mouse model of experimental autoimmune encephalomyelitis.^10^ These findings suggest that abnormality of GABA_A_ receptor function after SCI causes anxiety and locomotor dysfunction. However, the association between the administration of GABA_A_ agonist and functional recovery after SCI remains unclear. We hypothesized that ETZ inhibits neuroinflammation in the acute phase, reduces anxiety, and improves locomotor function after SCI. Therefore, this study investigated the effects of ETZ administration in the acute phase after SCI on neuroinflammation and behavioral function in mice.

## Methods

### Animals

In this study, we used 8-week-old male C57BL/6J mice weighing 18–20 g. Before the experimental procedure, we acclimatized animals to the captive environment for 5 days. Then, behavioral analysis was performed at the same time each day by a trained researcher blinded to treatment. We conducted all animal procedures following the guidelines of the Institutional Animal Care and Research Advisory Committee of Hirosaki University (approval no.: M20006-1).

### Spinal cord injury procedures

**FIG. 1.**
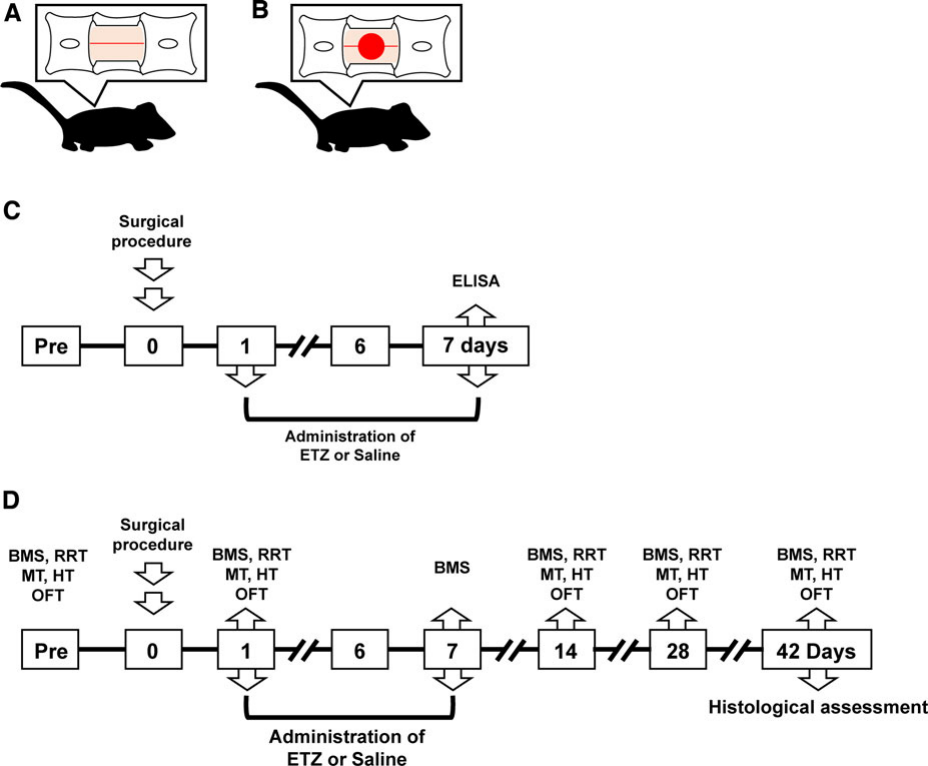
Experimental protocol. (**A**) Schematic diagram of laminectomy. A skin incision was made from the 9th to the 11th thoracic vertebrae, and a laminectomy was performed on the 10th thoracic vertebra. (**B**) Schematic diagram of SCI. After laminectomy, SCI was performed using an IH impactor. (**C**) Protocol Ⅰ. Collection of the spinal cord and measurement of inflammatory/anti-inflammatory cytokines were performed on day 7 after SCI. (**D**) Protocol Ⅱ. Mice were subjected to various behavioral tests, including locomotor function (BMS and RRT), sensory function (MT and HT), and anxiety-like behavior (OFT) assessments. BMS, Basso Mouse Scale; HT, heat test; MT, mechanical test; OFT, open field test; RRT, rotarod test; SCI, spinal cord injury.

Mice were anesthetized with an anesthetic mixture of 1% isoflurane and 30% oxygen. After shaving the skin, a scalpel blade was used to make a longitudinal incision along the dorsal skin surface. We induced SCI in the T10 after laminectomy at the 10th thoracic spinal vertebra (T10) to expose the spinal cord (Fig. 1A) by clamping the transverse processes of the T9 and T11 vertebrae (Fig. 1B) using a commercial available device (IH impactor; Precision Systems and Instrumentation, LLC, Lexington, KY), as described previously.^9,11^ This impactor uses a 2-mm impact rod tip to contuse the spinal cord and the user determines the impact force, defined by compression time or “dwell time.” We applied a force of 60 kilodynes (kdyn) with the impactor in the SCI groups.

### Etizolam treatment after spinal cord injury

We administrated 0.5 mg/kg of ETZ (Depas Tablets 0.5 mg; Mitsubishi Tanabe Pharma Corporation, Osaka, Japan) by daily intraperitoneal injection^13^ from the day after surgery for 7 days. We randomly divided the mice into three groups (sham group: only laminectomy; saline group: SCI + saline administration for 7 days; ETZ group: SCI + ETZ administration for 7 days).

### Experimental protocols

Two independent experimental protocols were used (Fig. 1C,D). In experimental protocol Ⅰ, the anti-inflammatory effect of ETZ was assessed in the acute phase after SCI (Fig. 1C). We collected spinal-cord samples on day 7 after surgery and measured cytokine concentrations (saline and ETZ groups: *n* = 9 for each group). In experimental protocol Ⅱ, behavior was assessed before surgery and on days 7, 14, 28, and 42 after surgery. All animals were habituated to the behavior test for several hours. On day 42 after surgery, all animals were perfused with paraformaldehyde, and a histological assessment was performed (sham, *n* = 11; saline, *n* = 9; ETZ, *n* = 11).

### Anti-inflammatory effects of etizolam in the acute phase

Concentrations of cytokines, including tumor necrosis factor alpha (TNF-α; BMS607HS; ThermoFisherScientific Inc., Waltham, MA), interleukin (IL)-1β (MLB00C; R&D Systems, Inc., Minneapolis, MN), and IL-10 (BMS614; ThermoFisherScientific), were measured in the spinal cord using the enzyme-linked immunosorbent assay (ELISA) by interpolation from a standard curve to investigate inflammatory/anti-inflammatory effects of ETZ in the acute phase after SCI. Spinal-cord samples were obtained from ∼10 mm spinal cord centered on the injured spinal cord in the saline and ETZ groups 7 days after surgery (Fig. 1C). Immediately after euthanization, lesioned spinal cord segments were carefully removed and homogenized (POLYTRON PT 10-35; Kinematica AG, Malters, Switzerland) with RIPA Lysis and Extraction Buffer, protease inhibitors, and PhosStop inhibitors. An equal amount of protein was subjected to ELISA, which was performed according to the manufacturer's instructions. Absorbance was measured on a plate reader (Crocodile 5-in-one; Titertek-Berthold, Pforzheim, Germany) at 620 nm. Results are expressed as pg/mL of protein.

### Assessment of anxiety-like behavior

As described previously, anxiety-like behavior was measured before surgery and on days 14, 28, and 42 after surgery (Fig. 1D).^9,11^ We first assessed anxiety-like behavior to avoid stressing the mice.

The open field test (OFT) is one of the most commonly used procedures to assess anxiety-like behavior and motor function in animal psychology and SCI research.^13^ Mice were individually placed in the open field arena (24 × 24 cm). Their exploratory behavior was recorded using a video tracking system (Capture Star; CleverSys, Inc., Reston, VA) for 10 min.^14^

When animals are first exposed to the OFT, it is possible to assess the emotional changes induced by exposure to the new environment.^14^ Mice tend to stay on the edge of the open field device in which they are placed. Emotionally impaired mice have less central arena entry than normal mice. Total distance traveled by the mouse and the percentage of walking distance in the middle 25% of the open field were calculated using analysis software (TopScan; CleverSys). Distance walked in the center 25% area divided by total distance walked was calculated as percentage of time spent in the center 25% (IC-25).^10^ A decrease in IC-25 value indicates an increase in anxiety. In addition, total walking distance was measured to evaluate locomotor functions.

IC-25 was normalized to pre-surgery and is expressed as a percentage of IC-25 post-surgery compared with pre-surgery because individual differences were observed pre-surgery. Therefore, the percentage of IC-25 post-surgery compared with the percentage pre-surgery was calculated as follows:


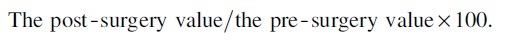


### Assessment of locomotor function

Locomotor function was measured before surgery and on days 14, 28, and 42 after surgery with the Basso Mouse Scale (BMS), the rotarod test, and total distance in the OFT, as described previously (Fig. 1D).^ 9,11^

The BMS is a useful tool for measuring locomotor function in SCI mice.^15^ The BMS for locomotor rating is a 10-point scale (0–9) based on the position of the mouse forelegs and trunk instability. In this study, two unbiased observers analyzed hindlimb performance using this score. The BMS score was recorded in mice by trained observers 7 days before surgery and on days 7, 14, 28, and 42 after surgery.

The rotarod (Rota Rod; Ugo Basile, Varese, Italy) test evaluates balance and coordination of the mice.^16,17^ In this test, the mouse is placed on a rotating rod, and the fall time is measured. The rotation was electronically set at a speed of 10 rpm. We set a maximum run time of 2 min. Three trials were performed for each mouse. Trained observers recorded the rotarod evaluations 7 days before surgery and on days 14, 28, and 42 after surgery.

### Assessment of sensory function

We also evaluated cutaneous sensitivity to mechanical and thermal stimulation as described previously (Fig. 1D).^**9**,1**1**^ Trained observers performed the mechanical and heat tests 7 days before surgery and on days 14, 28, and 42 after surgery.

In the mechanical test, mice were placed on an elevated wire mesh in plexiglass containers. The hindlimb plantar was pressed by filaments of mechanical strength using a commercially available device, and mechanical nociceptive thresholds for foot withdrawal were assessed. The reaction time required to lift the hindlimb by mechanical stimulation was measured (The Dynamic Plantar Aesthesiometer; Ugo Basile). We acclimated mice to the test area for 30 min before the test. Each test was performed five times on each hindlimb with a minimum test interval of 1 min and in a random order to minimize avoidance behavior.^18,19^

After the latency measurement, in the heat test, the Plantar Test Apparatus (Ugo Basile) was used to assess reactions to thermal stimulation. Mice are placed on an elevated glass surface in a plexiglass container in this test. When mice lift their paw, the photocell automatically turns off the heat source and timer. The thermal stimulation time was limited to a maximum of 20 sec, and the thermal stimulation was automatically discontinued to avoid tissue damage. Each test involved five trials on each hind paw, with at least 1-min intervals between trials, and the test was performed in a randomized order to minimize avoidance behaviors. We recorded latency (in seconds) to withdraw from the heat source.^20^

Reaction time was normalized to pre-surgery and is expressed as a percentage of reaction time post-surgery compared with pre-surgery because individual differences were observed pre-surgery. Therefore, the percentage of reaction time post-surgery compared with that pre-surgery was calculated as follows:


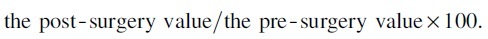


### Histological assessment

Histological assessment was used to investigate the anti-inflammatory effect of ETZ in the chronic phase after SCI. Spinal-cord samples were histologically evaluated using hematoxylin-eosin (HE) staining to determine atrophic change, Luxol fast blue (LFB) staining to determine demyelination, and immunohistochemistry to determine anti-inflammatory effects.^**9**,1**1**^ On day 42 after SCI, we anesthetized mice with isoflurane, followed by transdermal perfusion with saline, and 4% paraformaldehyde (0.1 M, pH 7.4), followed by incubation with a 30% sucrose solution (Fig. 1D). Spinal cords were removed, embedded in Optimal Cutting Temperature compound (Sakura FineTek Japan, Co., Ltd., Tokyo, Japan), and frozen. Frozen samples were cut into 20-μm-thick axial sections using a cryostat (Leica CM3050 S; Leica Microsystems GmbH, Wetzlar, Germany).

Stained spinal cords were analyzed using BZ-X700 software (Keyence, Osaka, Japan) to quantify HE- and LFB-stained areas. Areas of HE- or LFB-positive spinal-cord sections were measured and quantified in axial sections at the lesion epicenter at 40 × magnification using BZ-X700 software (Keyence).

For immunohistochemistry, tissue sections were incubated with the following primary antibodies: anti–glial fibrillary acidic protein (GFAP; ab4674; Abcam, Cambridge, MA) and anti–ionized calcium-binding adapter molecule 1 (Iba1; SC-28530; Santa Cruz Biotechnology Inc., Dallas, TX), followed by secondary antibodies (A11042 and A11055 Alexa Fluor; Thermo Fisher, Yokohama, Japan).^9^ Nuclei were stained with Hoechst 33258 and 4,6-diamidino-2-phenylindole. High-magnification photomicrographs were observed under a confocal microscope (CQ1 confocal image cytometer; Yokogawa Electric Corporation, Tokyo, Japan). Representative axial sections at the lesion epicenter were selected, and five regions were automatically captured in the epicenter at 400 × magnification (*n* = 5, each) to quantify the number of Iba1-positive cells and GFAP-positive areas. The number of Iba1-positive cells and GFAP-positive areas was quantified using the BZ-Analysis application (Keyence).

### Statistical analysis

All data are expressed as the mean ± standard error of the mean. Analyses were performed with SPSS software (Version 22; SPSS, Inc., Chicago, IL). Repeated measures of two-way analysis of variance (ANOVA), followed by the Tukey-Kramer test, was applied to compare behavioral assessments between pre- and post-surgery within and between groups. In addition, an unpaired *t*-test was applied to assess cytokine concentrations and histological findings. We considered a *p* value <0.05 to be statistically significant.

## Results

### Experimental protocol Ⅰ

**FIG. 2.**
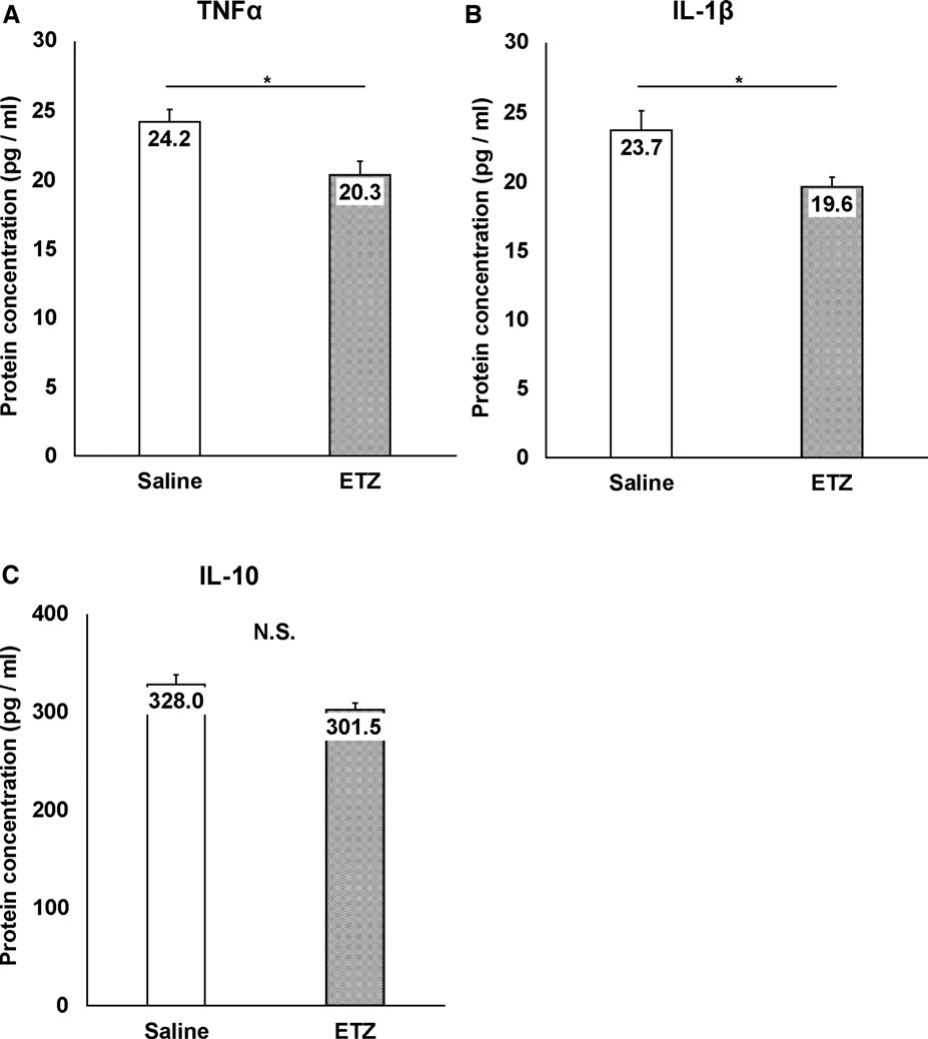
Anti-inflammatory effects of ETZ in the acute phase. (**A**) Comparison of TNF-α concentrations between the saline and ETZ groups on day 7 after injury. TNF-α concentrations were significantly lower after injury in the ETZ group than in the saline group (*n* = 9, each group). (**B**) Comparison of IL-1β concentrations between the saline and ETZ groups on day 7 after injury. IL-1β concentrations were significantly lower after injury in the ETZ group than in the saline group (*n* = 9, each group). (**C**) Comparison of IL-10 concentrations between the saline and ETZ groups on day 7 after injury. There was no significant difference in IL-10 concentrations between the saline and ETZ groups (*n* = 9, each group; *p < 0.05). ETZ, etizolam; IL-1β, interleukin-1β; IL-10, interleukin-10; TNF-α, tumor necrosis factor alpha.

Protein concentrations of inflammatory cytokines were significantly lower in the ETZ group than in the saline group (TNFα: 20.3 ± 0.9 vs. 24.2 ± 0.9, *p =* 0.014; IL-1β: 19.6 ± 0.7 vs. 23.7 ± 1.3, *p* = 0.022; Fig. 2A,B). In addition, the protein concentration of the anti-inflammatory cytokine (IL-10) was not different between the ETZ and saline groups (301.5 ± 7.4 vs. 327.9 ± 10.1, *p* = 0.064; Fig. 2C).

### Experimental protocol Ⅱ

#### Assessments of anxiety-like behavior

**FIG. 3.**
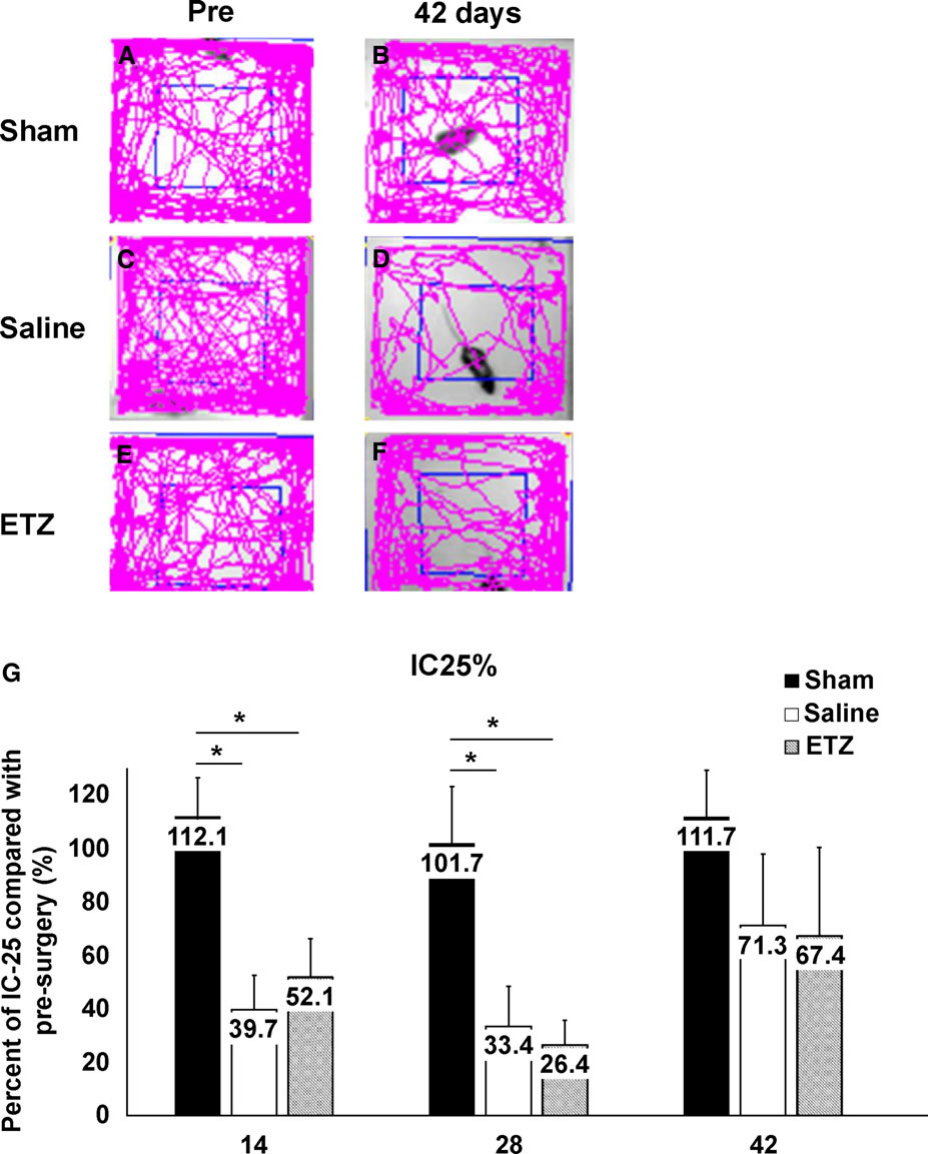
Assessment of anxiety-like behavior. (**A–F**) Representative open field test (OFT) walking traces of the sham, saline, and ETZ groups before and after injury. The center 25% of the field (IC-25%) is outlined in blue. (**G**) Comparison of OFT IC-25% among the three groups (sham, saline, and ETZ) at pre-injury and on days 14, 28, and 42 after injury. OFT IC-25% was significantly decreased on days 14 and 28 after injury in the saline and ETZ groups compared with the sham group. There was no significant difference between the saline and ETZ groups. *p < 0.05 versus each group, ANOVA. Sham: *n* = 11, saline: *n* = 9, and ETZ: *n* = 11. ANOVA, analysis of variance; ETZ, etizolam.

We evaluated the OFT IC-25 to investigate the effect of ETZ on anxiety-like behavior after SCI. All three groups showed similar behavior before surgery. The sham group showed no change in behavior after surgery compared with before surgery. However, the saline and ETZ groups showed decreased walking distance in the center after surgery compared to before surgery (Fig. 3A–F). In addition, fold increases in OFT IC-25 on days 14 and 28 were significantly lower in the saline and ETZ groups than in the sham group (14 days: sham: 89.2 ± 13.6%, saline: 45.7 ± 17.8%, and ETZ: 41.9 ± 17.2%; 28 days: sham: 78.6 ± 17.4%, saline: 12.3 ± 8.9%, and ETZ: 24.4 ± 12.3%; all *p* < 0.01; Fig. 3G). However, fold increases on days 14, 28, and 42 were not significantly different between the saline and ETZ groups (14 days, *p* = 0.83; 28 days, *p* = 0.96; 42 days, *p* = 0.99).

#### Assessments of locomotor function

We evaluated BMS, rotarod test performance, and OFT distance to investigate the effect of ETZ on locomotor function after SCI.

**FIG. 4.**
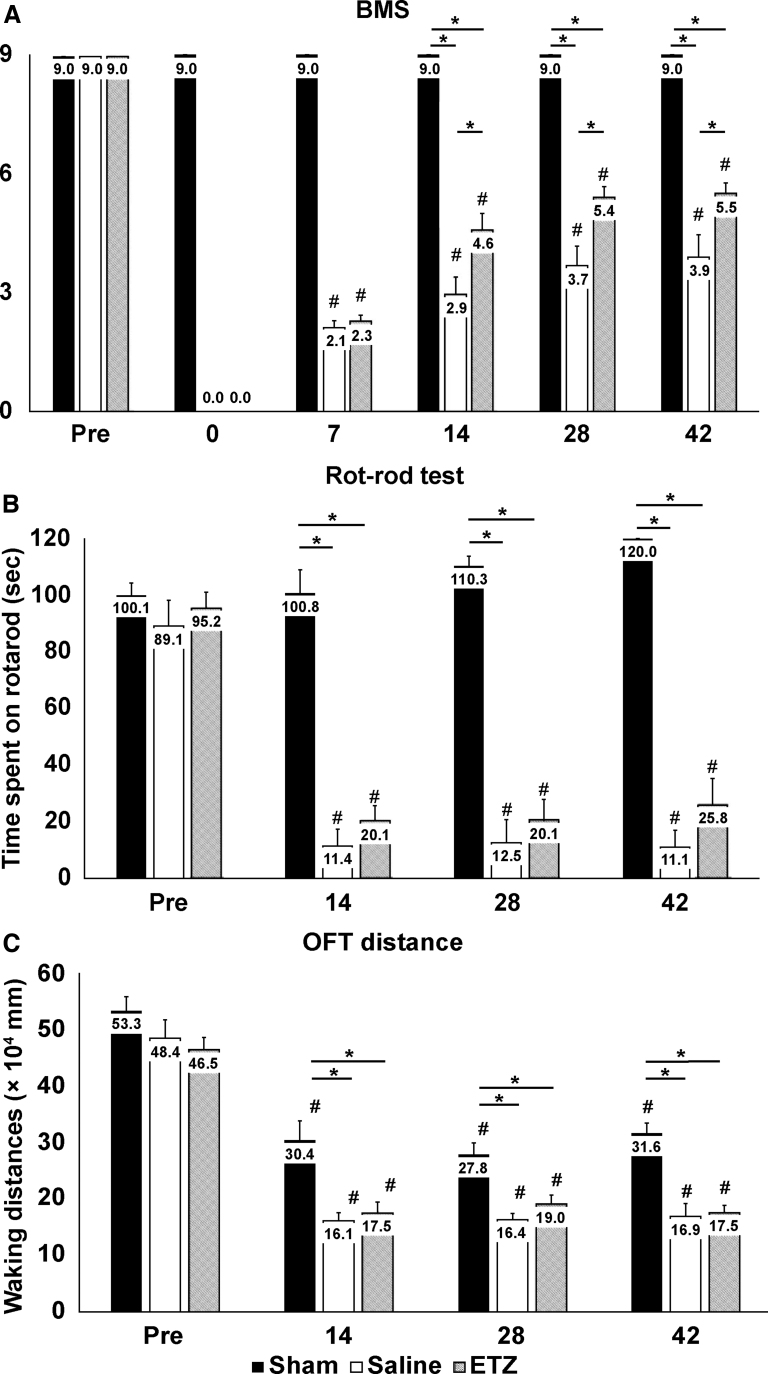
Assessment of motor functions. (**A**) Comparison of BMS scores among the three groups (sham, saline, and ETZ) at pre-injury and on days 7, 14, 28, and 42 after injury. BMS was significantly decreased after injury in the saline and ETZ groups compared with the sham group. BMS scores in the ETZ group were significantly higher than those in the saline group on days 14, 28, and 42 after injury. (**B**) Comparison of average riding times in the rotarod test among the three groups (sham, saline, and ETZ) at pre-injury and on days 14, 28, and 42 after injury. Average riding times in the rotarod test were significantly decreased after injury in the saline and ETZ groups compared with the sham group. There was no significant difference between the saline and ETZ groups. (**C**) Comparison of OFT distance among the three groups (sham, saline, and ETZ) at pre-injury and on days 14, 28, and 42 after injury. OFT distance was significantly decreased after injury in the saline and ETZ groups compared with the sham group. There was no significant difference between the saline and ETZ groups. ^#^*p* < 0.05 versus pre-injury, ANOVA. *p < 0.05 versus each group, ANOVA. Sham: *n* = 11, saline: *n* = 9, and ETZ: *n* = 11. ANOVA, analysis of variance; BMS, Basso Mouse Scale; ETZ, etizolam; OFT, open field test.

BMS scores on days 7, 14, 28, and 42 after surgery were significantly lower than those before surgery in the saline and ETZ groups (before surgery: saline: 9 and ETZ: 9; day 7: saline: 2.1 ± 0.2 and ETZ: 2.3 ± 0.1; day 14: saline: 2.9 ± 0.4 and ETZ: 4.5 ± 0.4; day 28: saline: 3.7 ± 0.5 and ETZ: 5.4 ± 0.3; day 42: saline: 3.9 ± 0.6 and ETZ: 5.5 ± 0.3; all *p* < 0.01). In addition, BMS scores did not change significantly on day 7 after surgery in the ETZ and saline groups. However, they improved significantly on days 14 (*p* < 0.01), 28 (*p* = 0.04), and 42 (*p* < 0.01) after surgery in the ETZ group (Fig. 4A).

In the rotarod test, average riding time on days 14, 28, and 42 after surgery was significantly shorter than that before surgery in the saline and ETZ groups (before surgery: saline: 101.0 ± 3.9 sec and ETZ: 88.0 ± 5.8 sec; day 14: saline: 3.9 ± 0.7 sec and ETZ: 18.9 ± 6.5 sec; day 28: saline: 4.2 ± 0.9 sec and ETZ: 20.1 ± 8.6 sec; day 42: saline: 6.1 ± 1.6 sec and ETZ: 27.6 ± 10.1 sec; all *p* < 0.01). However, no significant difference was noted in average riding time between the saline and ETZ groups after surgery (Fig. 4B).

OFT distances on days 14, 28, and 42 after surgery were significantly lower than those before surgery in the three groups (before surgery: sham: 5.3 ± 0.2 × 10^4^ mm, saline: 4.8 ± 0.3 × 10^4^ mm, and ETZ: 4.6 ± 0.2 × 10^4^ mm; day 7: sham: 3.0 ± 0.3 × 10^4^ mm, saline: 1.6 ± 0.1 × 10^4^ mm, and ETZ: 1.8 ± 0.2 × 10^4^ mm; day 28: sham: 2.8 ± 0.2 × 10^4^ mm, saline: 1.6 ± 0.1 × 10^4^ mm, and ETZ: 1.9 ± 0.2 × 10^4^ mm; day 42: sham: 3.2 ± 0.2 × 10^4^ mm, saline: 1.7 ± 0.2 × 10^4^ mm, and ETZ: 1.8 ± 0.1 × 10^4^ mm; all *p* < 0.01). No significant difference was noted in OFT distance between the saline and ETZ groups after surgery (Fig. 4C).

#### Assessments of sensory function

**FIG. 5.**
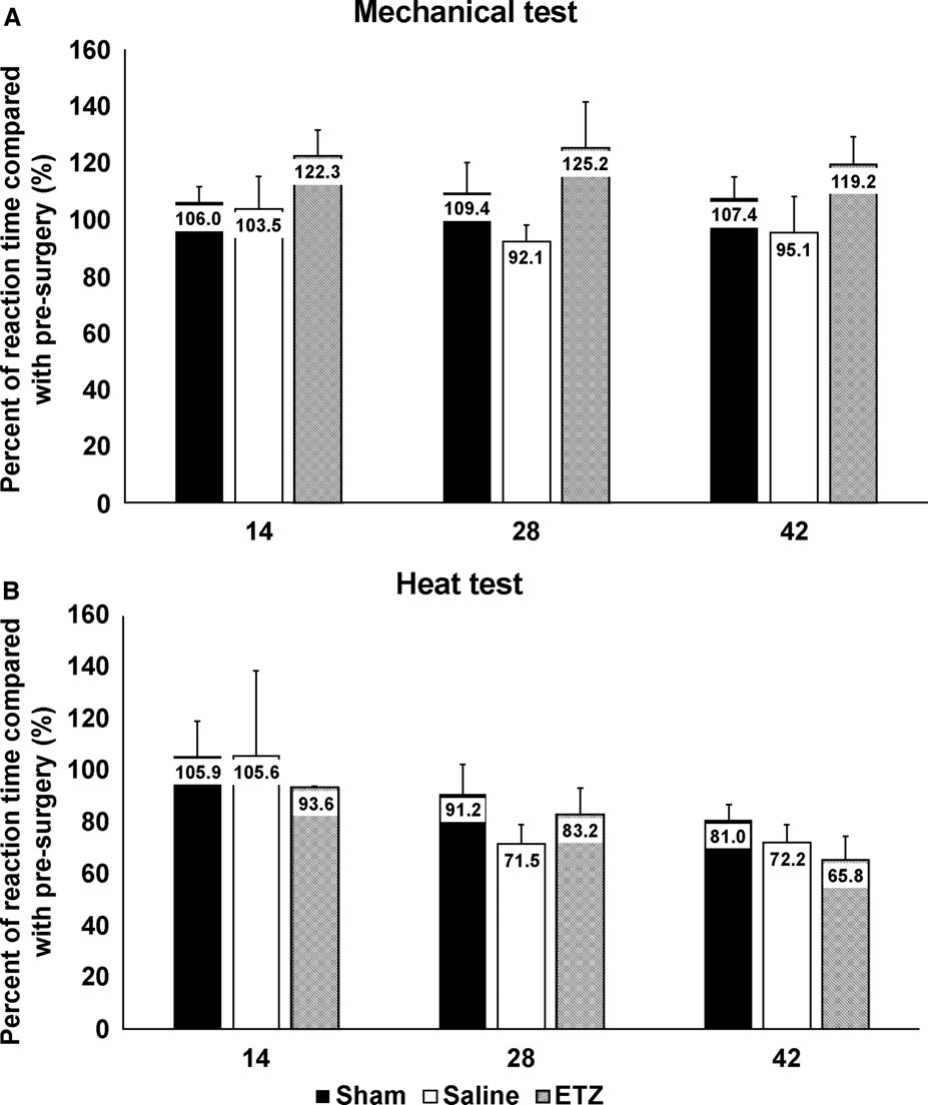
Assessment of sensory functions. (**A**) Comparison of fold increases in the mechanical test among the three groups (sham, saline, and ETZ) on days 14, 28, and 42 after surgery. (**B**) Comparison of fold increases in the heat test among the three groups. There was no significant difference between the three groups. Sham: *n* = 11, saline: *n* = 9, and ETZ: *n* = 11. ETZ, etizolam.

We used mechanical and heat tests to investigate the effect of ETZ on sensory function after SCI. In the mechanical and heat tests, fold increases on days 14, 28, and 42 after surgery were not significantly different among the three groups (Fig. 5A,B).

### Histological assessments

In the chronic phase, HE and LFB stainings and immunohistochemistry were used to assess atrophic change, demyelination, and anti-inflammatory effects, respectively.

**FIG. 6.**
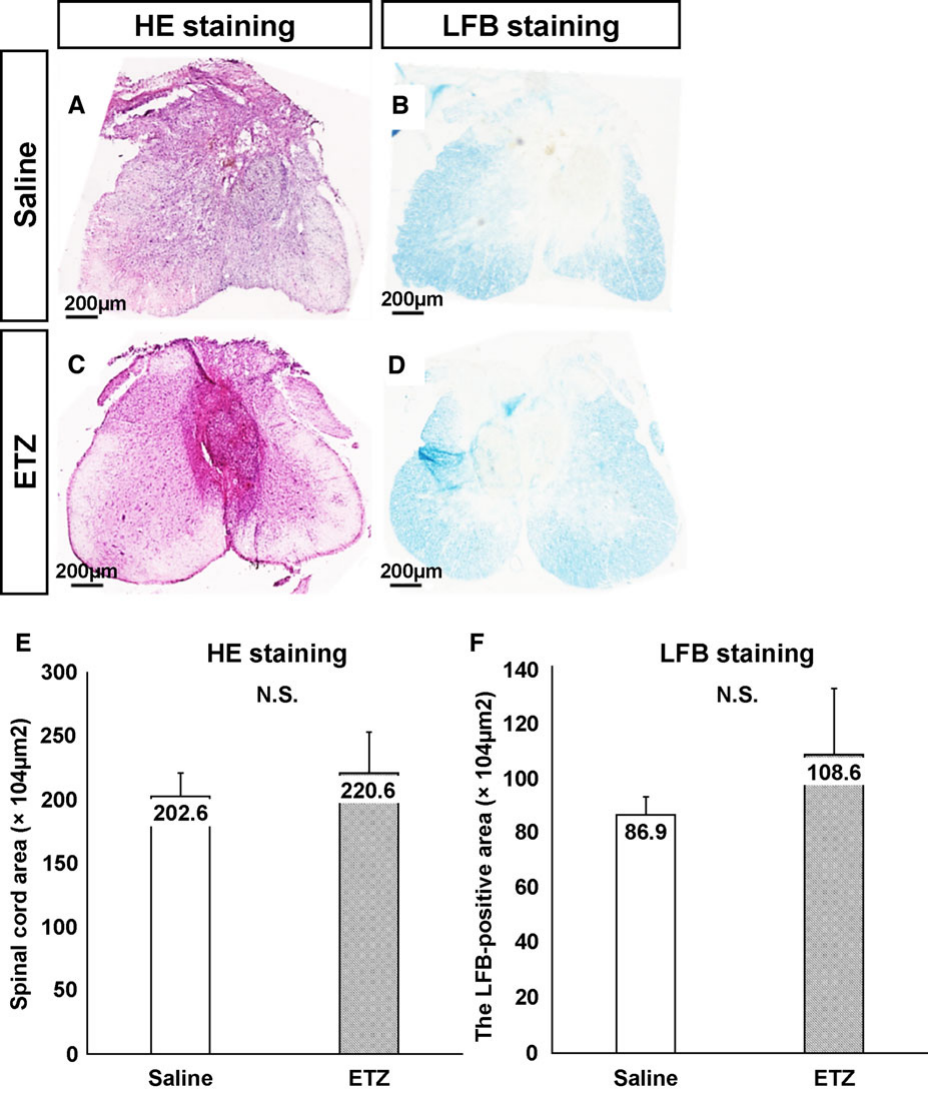
Histological assessments. (**A–D)** Spinal-cord atrophy and demyelination on day 42 after injury. Representative HE- and LFB-stained axial spinal-cord sections at the injury epicenter. Scale bars: 100 μm. (**E**) Area of HE staining at the lesion epicenter was not significantly different between the saline and ETZ groups (*n* = 5, each group). (**F**) Area of LFB staining at the lesion epicenter was not significantly different between the saline and ETZ groups (*n* = 5, each group). ETZ, etizolam; HE, hematoxylin-eosin; LFB, Luxol fast blue.

Atrophic changes and demyelination of injured spinal cords at the lesion epicenter were examined on day 42 after SCI by HE and LFB staining, respectively (Fig. 6A–D). No significant differences were noted in atrophic changes or demyelination in the transverse area of the spinal cord at the lesion epicenter between the saline and ETZ groups (Fig. 6E,F).

**FIG. 7.**
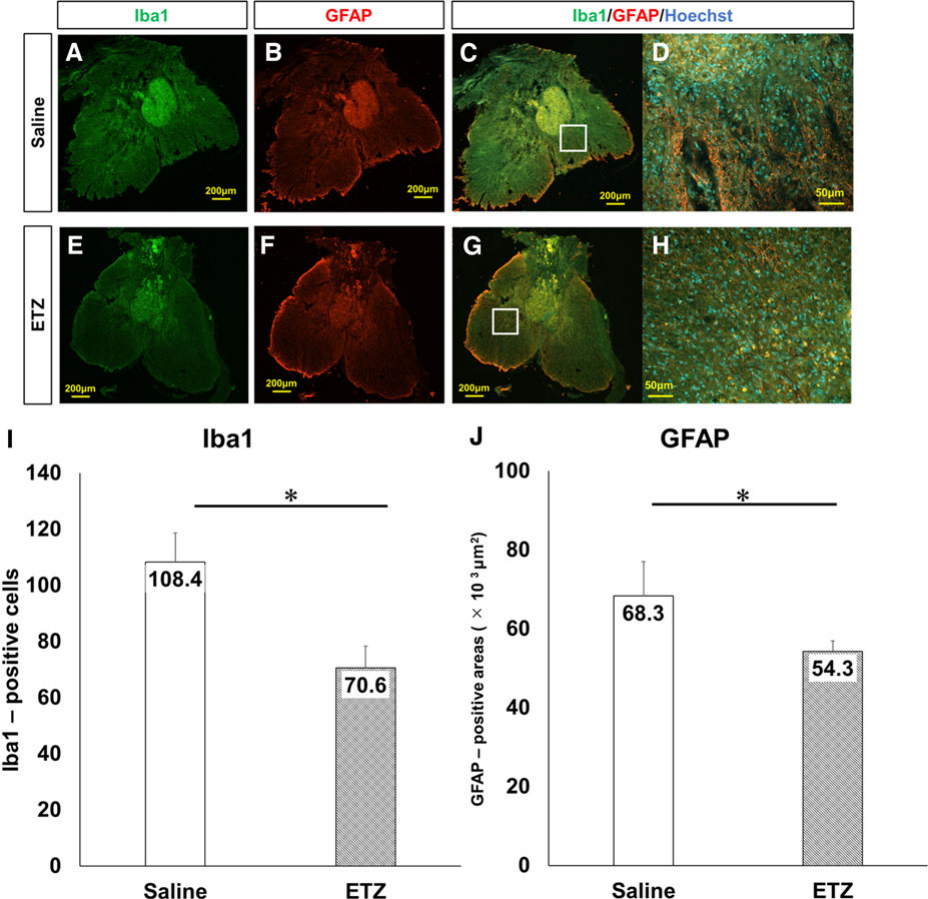
Anti-inflammatory effects of ETZ in the chronic phase. (**A–H**) Analysis of Iba1-positive microglial cells and GFAP-positive astrocytes at the lesion epicenter. Scale bars: 200 and 50 μm. More Iba1-positive microglial cells and GFAP-positive astrocytes were observed throughout the spinal cord in the saline group than in the ETZ group. High-magnification images (D) and (H) were obtained from (C) and (H), respectively. (**I**) Number of Iba1-positive microglia cells was significantly decreased in the ETZ group compared with the saline group (*n* = 5, each group; *p < 0.05). (**J**) Area of GFAP-positive astrocytes was significantly decreased in the ETZ group compared with the saline group (*n* = 5, each group; *p < 0.05). ETZ, etizolam; GFAP, glial fibrillary acidic protein; Iba1, ionized calcium-binding adapter molecule 1.

Inflammatory cell infiltration in the injured spinal cords was examined on day 42 after SCI by immunostaining for GFAP and Iba1. The ETZ group showed fewer inflammatory cells and GFAP-positive area at the lesion epicenter (Fig. 7A–H). In addition, the number of Iba1-positive cells and GFAP-positive areas in the center of the lesion was significantly decreased in the ETZ group compared with the saline group (Iba1: 70.6 ± 7.9 vs. 108.3 ± 10.3, *p* < 0.01; GFAP: 54.3 ± 2.6 × 10^3^ μm^2^ vs. 68.3 ± 8.7 × 10^3^ μm^2^, *p =* 0.022; Fig. 7I,J).

## Discussion

This study is the first to investigate the anti-inflammatory effects of ETZ administration after SCI in mice. Results showed that administration of ETZ in the acute phase after SCI reduced the concentration of inflammatory cytokines in the acute phase and the number of Iba1-positive microglia cells and GFAP-positive astrocyte areas in the chronic phase in the spinal cord. In addition, locomotor function improved in mice administrated ETZ, but sensory function and anxiety behavior did not.

Locomotor functional recovery might be attributable to the effect of an autoimmune reaction mediated by the GABA_A_ receptor activation induced by ETZ administration. SCI includes primary and secondary injury processes. Silva and colleagues reported that secondary injuries in the acute to subacute phases include disruption of the blood–spinal cord barrier, neuroinflammation, oxidative stress, neuronal damage, and ischemic dysfunction, which can lead to further tissue damage.^7^ They also demonstrated that neuroinflammation during the acute phase of SCI is caused by the generation of inflammatory cytokines from cells intrinsic to the CNS.^7^

In addition, other studies indicate that GABA_A_ receptors are associated with neuroinflammation in mice after SCI.^9^ After SCI, suppressing helper T cell 17 (Th17) differentiation inhibits IL-17 production and improves motor function.^21^ Moreover, it has been reported that administration of GABA_A_ agonists to experimental autoimmune encephalomyelitis mice suppresses Th17 cells by enhancing the response of regulatory T cells and inhibits the production of inflammatory cytokines in the CNS, thereby leading to anti-inflammatory effects.^10^ Further, a previous report demonstrated that the anti-inflammatory effects resulting by decreased values of various inflammatory cytokines such as TNF-α reduced glial scar formation after SCI.^22^ Therefore, the anti-inflammatory effect of ETZ on SCI reduced glial scar formation. In addition, these findings may improve locomotor function through GABAergic activity mediated by GABA_A_ receptors.

This study showed no significant differences in sensory function among the three groups. Consistently, some studies found that SCI mouse models, which were established with a force of 60 kdyn, did not show any significant differences in sensory function after SCI. ^9,11^ However, other studies observed that the same SCI mouse models had hypersensitivity to mechanical and thermal stimuli.^23^ Thus, the sensory function in the SCI mouse models, established with a force of 60 kdyn, needs further investigation. Notably, it has been reported that SCI mouse models established with a force of 50 kdyn are hypersensitive to mechanical and thermal stimuli and that treatment with rehabilitation improves their hypersensitivity.^24^ Therefore, it is possible that the administration of ETZ might improve sensory function in mice treated with a force of 50 kdyn.

It has been reported that anxiety behavior occurs after SCI in experimental animal models.^12^ Consistently, in this study, IC-25 value was significantly lower after SCI than before injury. GABA, a major inhibitory neurotransmitter in the CNS, mediates inhibition through GABA_A_ receptors,^25^ and thus GABAergic deficits in controlling emotionality contribute to anxiety disorders.^26^ Indeed, studies have shown that GABA receptor-mediated inhibition is reduced after SCI;^27^ mice with abnormal GABA_A_ receptors have more anxious behaviors than wild-type mice, and their anxious behaviors deteriorate after SCI.^9^ Although we hypothesized that ETZ would reduce anxiety behavior in the acute phase after SCI, ETZ administration did not improve anxiety behavior in any phase.

The reasons why ETZ did not improve anxiety behavior in our study's acute phase after SCI are explained below. First, it has been reported that a single dose of ETZ has a peak efficacy after 60 min and a half-life of 3–4 h, and the duration of the effect is ∼6 h.^8^ When anxiety-like behavior was assessed 30 min after administration, Narita and colleagues reported that acute administration of ETZ to the sciatic nerve ligation model mouse improved anxiety behavior.^28^ However, this study assessed anxiety behavior after the drug administration had been completed. Therefore, the anxiolytic effect of ETZ might have disappeared. Second, it has been reported that ETZ has a volume-dependent effect in rats in the dose range of 0.5–3.0 mg/kg.^29^ It has been reported that ETZ (1 mg/kg) administration to the sciatic nerve ligation model mouse from 7 to 21 days after injury improves anxiety-like behavior at 22 days after injury.^28^ In this study, we administered ETZ at a dose of 0.5 mg/kg in the acute phase for a week, which might not have been sufficient. Further studies with a higher dose of ETZ are needed to address our hypothesis.

This study has several limitations. First, we used only the IC-25 value for anxiety assessment. The OFT has been established as an anxiety assessment for the SCI mouse model.^12^ However, this assessment may be affected by locomotor disability after SCI. Therefore, developing a method to evaluate anxiety in mice unaffected by motor or sensory functions is necessary. Second, we described that ETZ to enhance GABAergic activity might affect Th17 cells associated with neuroinflammation in the CNS. However, the number of helper T cells in the CNS was not confirmed. Therefore, additional experiments with helper T cells and interleukin should be conducted. Third, cytokine levels in the brain have not been measured. It has been reported that inflammatory cytokines are elevated in the hippocampus of mice with anxiety behavior after SCI.^6^ GABA_A_ agonists have also been reported to have anti-inflammatory effects in the hippocampus.^10^ Therefore, additional experiments are needed to measure inflammatory cytokine levels in the brain, especially in the hippocampus.

Finally, we did not examine the effects of the long-term administration of ETZ. Given that dependence on long-term administration of ETZ has been considered problematic,^30^ the ideal treatment is short-term administration of ETZ. Despite these limitations mentioned above, this study is the first to demonstrate the anti-inflammatory effects of ETZ administration after SCI in mice.

## Conclusion

Administration of ETZ in the acute phase after SCI reduced neuroinflammation in the spinal cord. Locomotor function was improved in mice administrated ETZ, but sensory function and anxiety behavior did not. GABA_A_ receptor stimulants may be effective therapeutic agents in the acute phase after SCI.

## Abbreviations Used

ANOVA: analysis of variance
BMS: Basso Mouse Scale
CNS: sentral nervous system
ELISA: enzyme-linked immunosorbent assay
ETZ: etizolam
GABA: γ-aminobutyric acid
GABA_A_: γ-aminobutyric acid type A
GFAP: glial fibrillary acidic protein
HE: hematoxylin-eosin
Iba1: ionized calcium-binding adapter molecule 1
IC-25: percentage of time spent in the center 25%
IL: interleukin
kdyn: kilodynes
LFB: Luxol fast blue
OFT: open field test
SCI: spinal cord injury
Th17: helper T cell 17
TNFα: tumor necrosis factor alpha
